# The health system consequences of agency nursing and moonlighting in South Africa

**DOI:** 10.3402/gha.v8.26683

**Published:** 2015-05-11

**Authors:** Laetitia C. Rispel, Duane Blaauw

**Affiliations:** Centre for Health Policy & Medical Research Council Health Policy Research Group, School of Public Health, Faculty of Health Sciences, University of the Witwatersrand, Johannesburg, South Africa

**Keywords:** agency nursing, moonlighting, nurses, health system, quality of care, South Africa

## Abstract

**Background:**

Worldwide, there is an increased reliance on casual staff in the health sector. Recent policy attention in South Africa has focused on the interrelated challenges of agency nursing and moonlighting in the health sector.

**Objective:**

This paper examines the potential health system consequences of agency nursing and moonlighting among South African nurses.

**Methods:**

During 2010, a cluster random sample of 80 hospitals was selected in four South African provinces. On the survey day, all nurses providing clinical care completed a self-administered questionnaire after giving informed consent. The questionnaire obtained information on socio-demographics, involvement in agency nursing and moonlighting, and self-reported indicators of potential health system consequences of agency nursing and moonlighting. A weighted analysis was done using STATA^®^ 13.

**Results:**

In the survey, 40.7% of nurses reported moonlighting or working for an agency in the preceding year. Of all participants, 51.5% reported feeling too tired to work, 11.5% paid less attention to nursing work on duty, and 10.9% took sick leave when not actually sick in the preceding year. Among the moonlighters, 11.9% had taken vacation leave to do agency work or moonlighting, and 9.8% reported conflicting schedules between their primary and secondary jobs. In the bivariate analysis, moonlighting nurses were significantly more likely than non-moonlighters to take sick leave when not sick (*p*=0.011) and to pay less attention to nursing work on duty (*p*=0.035). However, in a multiple logistic regression analysis, the differences between moonlighters and non-moonlighters did not remain statistically significant after adjusting for other socio-demographic variables.

**Conclusion:**

Although moonlighting did not emerge as a statistically significant predictor, the reported health system consequences are serious. A combination of strong nursing leadership, effective management, and consultation with and buy-in from front-line nurses is needed to counteract the potential negative health system consequences of agency nursing and moonlighting.

Achieving universal health coverage to enable everyone to access the health services they need irrespective of ability to pay (1) and ensuring an adequately skilled, productive, and well-motivated health workforce (2) cannot be realised without addressing the global nursing crisis (3–5). This crisis is characterised by widespread shortages, an ageing workforce, excessive workloads, high turnover, skills gaps, and sub-optimal performance (3). The argument for addressing the nursing crisis is supported by well-documented evidence that the number, competencies, and effectiveness of nurses are critical in determining the quality of care and patient outcomes (6–16) and in improving the performance of weak health systems (4).

One aspect that has received inadequate attention in the description of the nursing crisis, by both the Global Health Workforce Alliance and the World Health Organization (WHO), is the casualisation of the nursing workforce and its implications for the nursing profession and for health system performance (2–4). The term ‘casualisation’ refers to the employment of workers on short-term contracts, without the rights and benefits associated with the standard contract of employment, namely full-time, permanent, continuing jobs (17). Although there are different types of casual or contingent work arrangements, the most visible form of casual work is through temporary staffing agencies (18) and moonlighting, defined as having a second job in addition to primary full-time employment. This policy gap exists despite increasing scholarly focus on the individual and organisational consequences associated with the greater reliance on casual or contingent staff in the workplace (17–26).

Research on casual work arrangements has focused *inter alia* on the commitment, roles, job satisfaction, conflict, perceived organisational support, organisational citizenship behaviours, health and well-being of contingent workers, and their performance in the workplace (18, 27). A review of research on casual or ‘precarious’ employment found an association between such employment and a deterioration in occupational health and safety in terms of injury rate and hazard exposures (25). Research on the performance of casual employees has yielded contradictory results, with their performance influenced by job satisfaction and commitment, type and scope of task allocation, and access to training (18).

A review of moonlighting among doctors in the health sector suggested that there are many negative health system consequences of this form of casualisation (28), including increased access barriers for patients, de-legitimisation of public sector health service delivery, reduction of trust between user and provider, lower quality of the care in the public sector, and accelerated migration to the private sector (28). In the case of agency nurses, there is evidence that casual or temporary staffing contributes to poor quality of patient care (29–32). In the United States, studies have found statistically significant associations between the employment of agency nurses and health care deficiencies in nursing homes (29), hospital medication errors (31), and the risk of bloodstream infections among patients with central venous catheters in intensive care units (32). In the United Kingdom, one study found that temporary staffing could undermine the quality of patient care (33), although another 5-year study of general and specialist wards found no differences in quality scores between temporary and permanent nursing staff (34).

Although several authors have highlighted the importance of understanding casualisation in low- and middle-income countries, particularly in the health sector (17, 28, 35, 36), much of the existing literature is concerned with high-income countries (18, 19, 22, 37–39). In South Africa, recent health policy attention has focused on the twin challenges of agency nursing and moonlighting (40, 41). A 2010 cross-sectional study found that agency nursing and moonlighting – two manifestations of casualisation in nursing – were common (42). The occurrence of moonlighting among nurses in the 12 months preceding the survey was 28.0% and of agency nursing was 37.8% (42). In light of the importance of nurses to improving the performance of the South African health system, this paper examines the potential health system consequences of agency nursing and moonlighting among South African nurses, using data from the same survey. The findings of the study are part of a larger research project to examine casualisation in the nursing profession.

## Methods

During 2010, a one-stage cluster random sample of 80 hospitals was selected from the four South African provinces of the Eastern Cape (mixed urban-rural). Free State (mixed urban-rural), Gauteng (urban), and the Western Cape (predominantly urban). The Human Research Ethics Committee (Medical) of the University of the Witwatersrand in Johannesburg provided ethics approval for the study. The relevant public and private health care authorities also provided the necessary study approvals. All participants provided written, informed consent.

In each of the four provinces, the sampling frame consisted of all public and private hospitals, stratified by type of hospital for public hospitals and by ownership and hospital bed numbers for private hospitals. A random sample of public and private sector hospitals was then selected from each stratum proportional to the total number of hospitals in that stratum.

In the study, moonlighting was defined as additional paid work – whether of a nursing or non-nursing nature – done by nurses in a private health facility, another government health facility, an insurance company, private health laboratory, or in the same health care facility while holding a primary, paid nursing job, but excluding overtime (42). Agency nursing was defined as any accredited nurse providing temporary cover in a hospital and paid for by a commercial nursing agency (42).

On the 24-hour survey day, all nurses working in critical care, theatre, emergency, maternity, and general medical and surgical wards completed a self-administered questionnaire after giving informed consent. The questionnaire obtained information on demographic characteristics, agency nursing and moonlighting, and participants’ experiences of health system incidents in the 12 months preceding the survey. These incidents are proxy indicators of health system consequences of agency nursing and moonlighting, identified in the international literature and in the formative research conducted prior to the survey. All participants were asked to indicate whether they had felt too tired to work while on duty; paid less attention to nursing work while on duty; taken sick leave when not sick; stayed away from work without authority to do so; or been involved in a medico-legal incident, for example administration of the wrong medication or a patient death (type 1 indicators). In addition, those participants who indicated that they had done moonlighting or agency nursing in the 12 months preceding the survey were asked to indicate whether they had argued with doctors or other nurses, experienced conflicting schedules between their primary and secondary jobs, taken sick leave to do agency work or moonlighting, taken vacation leave to do agency work or moonlighting, stayed away from work without authority to do agency work or moonlighting, or treated patients differently (e.g. shouted at patients) in their primary compared to their secondary job (type 2 indicators). Further details of the survey methodology are provided in a previous article (42).

Data were weighted to reflect the population distribution of nurses between the public and private health sectors and the four study provinces; and analysed using STATA^®^ 13. We also adjusted for the clustering and stratification introduced by the sampling design. Frequency tabulations were done to describe the socio-demographic characteristics of the respondents. Cross-tabulations were done to investigate associations of each of the factors, including agency nursing and moonlighting, with the type 1 and type 2 proxy indicators of health system consequences in the 12 months preceding the survey, our main outcomes of interest. Bivariate logistic regression models were fitted and only factors found to be statistically significantly associated with the health system consequences at a conservative 20% level were considered further in the final model-building process using multiple logistic regression. All other statistical tests were considered significant at the 5% level.

We also used multiple correspondence analysis (MCA) to derive two indices of health system consequences (for type 1 and type 2 indicators). MCA is a data reduction method similar to principal component analysis but more appropriate for categorical data (43, 44). The MCA index was normalised to a mean of zero and a standard deviation of 1, hence positive scores indicate more adverse health system consequences. Bivariate differences in the consequence indices were tested by *t*-tests and ANOVA.

## Results

### Participant characteristics

The majority of survey participants were female (92.7%) and employed in provincial government (52.8%). The participants were predominantly middle-aged, with a mean age of 41.5 (SD: 10.4) years. The unweighted demographic and background characteristics of the 3,784 nurses recruited in the four study provinces are shown elsewhere in this special journal issue (45). Importantly, 40.7% (95% CI: 35.3–46.4) of nurses indicated that they had moonlighted or worked for an agency in the 12 months prior to the survey.

### Occurrence of health system consequences

In the study, 51.5% of all participants said that they felt too tired to work while on duty, 11.5% paid less attention to nursing work while on duty, 10.9% had taken sick leave when not actually sick, 5.6% had stayed away from work without authority, and 2.9% reported being involved in a medico-legal incident (Table 1). More than half (55.8%) of nurses were involved in any of these incidents used as proxy indicators of health system consequences. The mean of the MCA composite index combining these variables was 0.761, indicating that on average there were more undesirable consequences.

**Table 1:** Health system consequences of moonlighting and agency work

			Felt too tired to work while on duty		Paid less attention to nursing work while on duty		Took sick leave when not actually sick		Stayed away from work without authority		Involved in a medico-legal incident		Any of these		Composite Consequence Index 1 (MCA*)	
									
Variable		*n*	%	*p*	%	*p*	%	*p*	%	*p*	%	*p*	%	*p*	Mean	*p*
Total		3708	51.5		11.5		10.9		5.6		2.9		55.8		0.761	
Moonlighting or agency	No	2143	52.2	0.442	10.3	**0.035**	9.7	**0.011**	4.9	0.090	2.6	0.169	56.5	0.463	−0.018	0.138
nursing	Yes	1473	50.5		13.2		12.9		6.5		3.4		55.0		0.032	
Province	Gauteng	1638	55.6	**0.005**	13.6	**0.049**	13.2	**0.018**	6.9	**0.012**	3.1	0.299	59.5	**0.007**	0.197	**<0.001**
	Eastern Cape	945	54.6		10.6		10.4		6.0		2.4		59.4		0.001	
	Western Cape	800	44.2		9.2		9.3		3.3		2.7		48.1		−0.102	
	Free State	325	40.5		9.4		5.5		4.3		4.4		45.6		−0.135	
Sex	Male	289	53.4	0.621	14.4	0.135	14.8	0.090	6.7	0.416	3.5	0.579	58.6	0.447	0.230	<**0.001**
	Female	3412	51.3		11.2		10.6		5.5		2.9		55.5		−0.013	
Age group	<25 years	139	54.5	<**0.001**	14.3	**0.017**	14.8	<**0.001**	7.2	<**0.001**	4.0	0.290	60.4	<**0.001**	0.183	<**0.001**
	25–34 years	926	59.4		15.1		16.6		9.5		4.0		63.9		0.235	
	35–44 years	1024	52.4		11.0		11.0		5.2		2.9		56.6		0.026	
	45–54 years	1060	50.0		10.4		8.9		4.4		2.3		54.4		−0.094	
	55+ years	430	35.8		8.3		3.5		1.7		1.9		40.3		−0.315	
Marital status	Married/living together	1667	51.9	**0.011**	11.5	0.513	11.0	0.231	4.8	<**0.001**	3.2	0.509	56.5	**0.004**	−0.021	**0.007**
	Single	1495	53.3		10.9		11.6		7.4		2.4		57.7		0.071	
	Divorced/widowed	530	44.7		13.1		8.2		3.2		3.3		47.9		−0.070	
Any children?	No	619	56.6	0.084	15.6	**0.012**	13.2	0.099	4.9	0.457	4.1	0.129	62.3	**0.016**	0.120	**0.002**
	Yes	3089	50.5		10.7		10.5		5.8		2.7		54.5		−0.019	
Sector	Public	2692	54.3	<**0.001**	11.0	**0.012**	11.5	**0.011**	5.8	**0.002**	2.4	**0.041**	58.3	<**0.001**	0.078	<**0.001**
	Private	681	46.5		15.1		7.1		2.9		4.2		51.9		−0.075	
	Agency	259	35.3		8.7		12.7		9.1		3.6		39.7		−0.113	
Nursing category	Professional nurse	1759	57.4	<**0.001**	15.2	<**0.001**	12.3	0.134	5.5	0.526	3.9	**0.010**	61.5	<**0.001**	0.110	<**0.001**
	Enrolled nurse	740	52.1		9.1		10.6		6.6		2.2		56.3		−0.038	
	Nursing assistant	1209	42.3		7.4		9.1		5.2		2.0		47.0		−0.162	
Unit	General wards	1160	51.3	0.482	12.1	0.134	11.1	0.962	6.1	0.258	3.1	0.632	56.1	0.305	−0.007	0.355
	Maternity	599	52.5		8.9		11.2		4.2		2.8		55.7		−0.044	
	ICU	536	50.5		14.7		11.8		4.1		3.2		56.4		0.062	
	Theatre	570	55.2		11.6		10.1		7.5		1.9		59.7		0.039	
	Other	834	49.1		10.5		10.6		5.7		3.1		52.3		−0.012	
Years working at	Less than 1 year	404	47.2	0.257	10.7	0.146	8.4	**0.005**	4.8	0.389	2.3	**0.005**	50.6	0.310	−0.047	<**0.001**
primary job	1–4 years	982	53.2		10.3		11.6		6.9		4.0		57.4		0.054	
	5–9 years	675	55.3		14.5		15.8		6.3		2.1		59.2		0.097	
	10–14 years	339	54.9		13.1		13.4		4.5		4.6		58.5		0.037	
	15–19 years	312	53.0		13.5		8.4		3.8		4.6		57.9		−0.029	
	20 or more years	904	49.0		10.2		7.6		4.9		1.3		54.3		−0.106	

Type 1 indicators in all nurses; Statistically significant relationships in bold.

* Multiple correspondence analysis.

Table 1 compares the occurrence of health system consequences between nurses who had moonlighted or worked for an agency in the preceding year and those who had not. Moonlighting nurses were significantly more likely to take sick leave when not sick (12.5% vs. 9.7%; *p*=0.011) and to pay less attention to nursing work while on duty (13.2% vs. 10.3%, *p*=0.035), but there were no significant differences between the two groups for any of the other type 1 indicators. The MCA composite index was higher for moonlighting nurses but again the difference was not statistically significant. Table 1 also shows the bivariate analysis of other socio-demographic factors associated with these outcomes. Significant differences were noted for different individual outcomes and for the composite index between different provinces, age groups, sectors of work, and nursing categories.

Type 2 indicators were only collected among those participants that had done moonlighting or agency nursing in the year preceding the survey (Table 2). In this group, 19.6% reported that they argued with their colleagues, 9.8% reported conflicting schedules between their primary and secondary jobs, but only 2.3% reported that they treated patients differently in the primary compared to the secondary job. In addition, 11.9% reported that they had taken vacation leave and 2.8% had taken sick leave to do agency work or moonlighting, but only 1.6% indicated that they stayed away from work without authority to moonlight or work for an agency in the preceding year. One-third of moonlighters (33.7%) reported any of these type 2 indicators, and the average score of the MCA composite index derived from these variables was positive at 0.422. Again the bivariate analysis suggested differences for certain of these outcomes between provinces, age groups, sector of work, and nursing category (Table 2).

**Table 2:** Health system consequences of moonlighting and agency work

			Argued with doctors or other nurses		Conflicting schedules between primary and secondary jobs		Took vacation leave to do agency work or moonlighting		Took sick leave to do agency work or moonlighting		Stayed away without authority to do agency work or moonlighting		Treated patients differently in primary vs. secondary job		Any of these		Composite Consequence Index 2 (MCA)	
										
Variable		*n*	%	*p*	%	*p*	%	*p*	%	*p*	%	*p*	%	*p*	%	*p*	Mean	*p*
Total		1473	19.6		9.8		11.9		2.8		1.6		2.3		33.7		0.422	
Province	Gauteng	897	23.2	<**0.001**	12.1	<**0.001**	13.9	**0.011**	3.2	0.204	2.4	0.475	3.4	**0.002**	38.5	<**0.001**	0.218	<**0.001**
	Eastern Cape	122	24.1		6.4		11.2		2.8		0.0		0.2		34.3		−0.102	
	Western Cape	334	11.4		4.3		6.5		2.7		0.0		0.6		20.7		−0.205	
	Free State	120	12.1		11.9		13.9		0.6		1.8		1.7		34.7		−0.047	
Sex	Male	107	25.3	**0.042**	13.2	0.188	16.8	0.140	1.6	0.372	2.1	0.579	1.2	0.459	39.9	0.211	0.219	**0.015**
	Female	1362	19.1		9.6		11.6		2.9		1.6		2.4		33.2		−0.014	
Age group	<25 years	51	15.0	0.651	8.8	0.397	2.4	0.070	3.5	0.521	3.4	0.500	3.5	0.718	22.9	0.123	0.036	**0.035**
	25–34 years	402	21.6		10.7		11.2		3.8		2.0		3.3		36.4		0.039	
	35–44 years	463	20.1		11.9		15.4		2.7		1.3		2.2		37.2		0.075	
	45–54 years	377	18.1		7.2		11.3		2.7		0.9		1.5		29.6		−0.080	
	55+ years	114	16.2		9.9		8.6		0.0		0.0		3.5		28.7		−0.181	
Marital status	Married/living together	648	19.1	0.971	9.7	0.523	12.3	0.336	3.0	0.661	1.7	0.978	1.8	0.247	32.9	0.541	−0.011	0.832
	Single	613	19.8		9.1		10.8		2.9		1.6		2.3		33.2		0.002	
	Divorced/widowed	199	19.7		11.7		14.9		1.5		1.5		4.4		37.7		0.033	
Any children?	No	232	22.2	0.305	9.6	0.886	10.9	0.623	2.2	0.592	1.2	0.671	2.0	0.751	33.9	0.920	−0.023	0.657
	Yes	1241	19.0		9.9		12.1		2.9		1.7		2.4		33.6		0.007	
Sector	Public	823	20.1	0.134	9.9	0.916	12.7	**0.001**	3.1	0.191	1.2	**0.004**	3.4	0.151	34.8	**0.019**	0.116	**0.001**
	Private	413	21.1		9.3		15.1		1.7		0.7		0.9		35.8		−0.091	
	Agency	203	12.6		9.8		2.2		3.7		6.0		1.5		22.6		0.017	
Nursing category	Professional nurse	735	22.7	0.068	9.5	0.543	17.9	<**0.001**	2.5	0.114	1.2	0.547	2.7	0.284	38.7	**0.003**	0.049	0.109
	Enrolled nurse	315	15.2		11.5		8.3		4.5		1.9		1.0		27.9		−0.024	
	Nursing assistant	423	17.0		9.1		3.7		2.0		2.2		2.8		28.7		−0.081	
Unit	General wards	386	17.7	0.239	10.2	0.167	6.3	<**0.001**	3.3	0.605	1.8	0.290	1.6	0.296	29.3	**0.009**	−0.043	**0.014**
	Maternity	274	18.0		6.5		6.9		1.4		0.0		2.1		25.6		−0.139	
	ICU	372	17.9		11.1		20.9		3.7		2.1		3.3		39.3		0.129	
	Theatre	207	26.3		14.8		12.1		2.0		1.2		3.7		43.0		0.031	
	Other	234	21.3		6.7		12.9		3.1		2.8		1.1		33.5		0.001	
Years working at	Less than 1 year	180	12.6	0.186	10.3	0.428	8.6	**0.047**	5.0	0.154	2.0	0.360	2.6	0.240	27.6	0.157	−0.011	0.878
primary job	1–4 years	448	21.8		7.8		8.7		3.4		2.9		3.0		32.3		0.028	
	5–9 years	290	18.7		12.4		13.7		2.2		0.9		0.9		36.8		−0.023	
	10–14 years	170	21.9		12.4		17.7		4.6		1.2		1.7		39.1		0.070	
	15–19 years	125	24.7		9.1		18.1		0.0		0.0		1.1		41.5		−0.038	
	20 or more years	205	19.0		7.7		13.4		0.7		0.7		4.8		32.6		−0.021	

Type 2 indicators in moonlighting nurses only; Statistically significant relationships in bold.

### Predictors of negative health system consequences among all participants

Table 3 shows the results of the multiple regression analysis used to investigate the impact of moonlighting on the type 1 health system consequences, while adjusting for other socio-demographic factors. Among moonlighters, the odds of “staying away from work without authority” were 1.31 (95% CI: 0.86–1.98) times higher than for non-moonlighters, and the odds of “taking sick leave when not actually sick” were 1.36 (95% CI: 0.95–1.94) higher, but these differences were only statistically significant at the 10% level in the multiple regression. Moonlighters were 1.19 (95% CI: 0.88–1.62) times more likely to report “paying less attention to nursing work while on duty,” which was also not statistically significant. The odds of “being involved in a medico-legal incident” and “feeling too tired at work” were similar in the two groups. Overall, experiencing any of these incidents was equally likely in the two groups (OR: 0.99; 95% CI: 0.80–1.20).

**Table 3:** Multiple logistic regression of predictors of health system consequences

		Felt too tired to work while on duty	Paid less attention to nursing work while on duty	Took sick leave when not actually sick	Stayed away from work without authority	Involved in a medico-legal incident	Any of these
							
Variable		OR (95% CI)	OR (95% CI)	OR (95% CI)	OR (95% CI)	OR (95% CI)	OR (95% CI)
Moonlighting or	No	–	–	–	–	–	–
agency nursing in	Yes	0.97	1.19	1.36	1.31	1.01	0.99
the past 12 months		(0.79–1.19)	(0.88–1.62)	(0.95–1.94)	(0.86–1.98)	(0.63–1.62)	(0.81–1.20)
Province	Gauteng	–	–	–	–		–
	Eastern Cape	0.95	0.87	1.02	1.03		1.02
		(0.60–1.51)	(0.59–1.28)	(0.72–1.45)	(0.65–1.63)		(0.64–1.62)
	Western Cape	0.65**	0.67**	0.84	0.54***		0.64**
		(0.49–0.85)	(0.52–0.85)	(0.53–1.36)	(0.40–0.75)		(0.49–0.83)
	Free State	0.58***	0.71*	0.44***	0.74		0.60***
		(0.42–0.78)	(0.51–0.98)	(0.29–0.68)	(0.49–1.11)		(0.45–0.80)
Sex	Male		–	–			
	Female		0.78	0.76			
			(0.50–1.21)	(0.47–1.23)			
Age group	<25 years	–	–	–	–		–
	25–34 years	1.32	1.10	0.88	1.30		1.27
		(0.77–2.27)	(0.63–1.91)	(0.46–1.65)	(0.66–2.55)		(0.83–1.95)
	35–44 years	0.94	0.62	0.45*	0.69		0.89
		(0.55–1.61)	(0.34–1.13)	(0.24–0.87)	(0.32–1.48)		(0.57–1.39)
	45–54 years	0.83	0.60	0.37*	0.71		0.81
		(0.47–1.49)	(0.35–1.02)	(0.18–0.79)	(0.31–1.60)		(0.51–1.29)
	55+ years	0.47*	0.45*	0.15***	0.19*		0.46**
		(0.24–0.91)	(0.22–0.93)	(0.06–0.38)	(0.04–0.97)		(0.27–0.80)
Marital status	Married/living together	–			–		–
	Single	0.95			1.21		0.93
		(0.79–1.13)			(0.83–1.78)		(0.78–1.11)
	Divorced/widowed	0.99			0.84		0.94
		(0.77–1.26)			(0.48–1.48)		(0.74–1.20)
Any children?	No	–	–	–		–	–
	Yes	0.86	0.79	0.94		0.65	0.78
		(0.63–1.16)	(0.57–1.11)	(0.64–1.38)		(0.35–1.19)	(0.58–1.03)
Sector	Public	–	–	–	–	–	–
	Private	0.68**	1.30*	0.49**	0.50*	1.32	0.73*
		(0.53–0.89)	(1.02–1.66)	(0.32–0.75)	(0.30–0.86)	(0.79–2.21)	(0.57–0.94)
	Agency	0.50***	0.79	1.27	1.40	1.56	0.50***
		(0.38–0.66)	(0.48–1.31)	(0.80–1.99)	(0.82–2.40)	(0.80–3.04)	(0.37–0.67)
Nursing category	Professional nurse	–	–	–		–	–
	Enrolled nurse	0.77	0.57**	0.73		0.58	0.77
		(0.56–1.04)	(0.39–0.83)	(0.52–1.04)		(0.31–1.06)	(0.58–1.04)
	Nursing assistant	0.52***	0.44***	0.71		0.50*	0.54***
		(0.41–0.66)	(0.30–0.65)	(0.49–1.04)		(0.26–0.93)	(0.42–0.69)
Unit	General wards		–				
	Maternity		0.63*				
			(0.43–0.93)				
	ICU		0.91				
			(0.62–1.33)				
	Theatre		0.89				
			(0.63–1.25)				
	Other		0.82				
			(0.56–1.20)				
Years working at	Less than 1 year		–	–		–	
primary job	1–4 years		0.93	1.57*		1.82	
			(0.63–1.37)	(1.00–2.46)		(0.90–3.68)	
	5–9 years		1.45	2.88***		0.95	
			(0.97–2.16)	(1.80–4.63)		(0.38–2.38)	
	10–14 years		1.40	2.71**		1.89	
			(0.89–2.20)	(1.43–5.12)		(0.69–5.16)	
	15–19 years		1.56	1.79		2.14	
			(0.94–2.60)	(0.85–3.79)		(0.80–5.76)	
	20 or more years		1.45	2.00		0.58	
			(0.89–2.37)	(0.88–4.55)		(0.21–1.59)	
Constant		2.36**	0.38*	0.21***	0.07***	0.04***	3.09***
		(1.42–3.94)	(0.17–0.82)	(0.10–0.47)	(0.03–0.21)	(0.02–0.09)	(2.16–4.44)
Observations		3357	3277	3281	3354	3401	3375
Model *p* value		<0.001	<0.001	<0.001	<0.001	<0.001	<0.001

Type 1 indicators in all nurses.

*** *p*<0.001,

** *p*<0.01,

* *p*<0.05.

Instead, the multiple logistic regression analysis found that the differences for these variables were explained by province (geographical location), age, sector of employment, nursing category, and the number of years at the primary job (Table 3).

In terms of geographical location and relative to Gauteng, those participants from the Western Cape were significantly less likely to report that they stayed away from work without authority (OR: 0.54; 95% CI: 0.40–0.75); felt too tired to work while on duty (OR: 0.65; 95% CI: 0.49–0.85); or paid less attention to nursing work while on duty (OR: 0.67; 95% CI: 0.52–0.85). Participants from the Free State province were also less likely to report that they felt too tired to work while on duty, took sick leave when not actually sick, or paid less attention to nursing work while on duty (Table 3).

Interestingly, those nurses over 55 years old were much less likely to report that they stayed away without authority to do so (OR: 0.19; 95% CI: 0.04–0.97); felt too tired to work while on duty (OR: 0.47; 95% CI: 0.24–0.91); had taken sick leave when not actually sick (OR: 0.15; 95% CI: 0.06–0.38); or paid less attention to nursing work while on duty (OR: 0.45; 95% CI: 0.22–0.93).

Relative to the public sector participants, those nurses from the private sector were less likely to report that they stayed away from work without authority (OR: 0.50; 95% CI: 0.30–0.86), felt too tired to work while on duty (OR: 0.68; 95% CI: 0.53–0.89); or had taken sick leave when not actually sick (OR: 0.49; 95% CI: 0.32–0.75). However, they were more likely to report that they paid less attention to nursing while at work (OR: 1.30; 95% CI: 1.02–1.66). Those from a commercial nursing agency were also less likely to report that they felt too tired to work while on duty (OR: 0.50; 95% CI: 0.38–0.66).

The analysis found that nursing assistants were less likely than professional nurses to report any of the incidents measured in the study. However, there were differences related to the number of years of employment at the primary job, with nurses who had been working for between 1 and 14 years reporting higher likelihoods of taking sick leave when not actually sick (Table 3).

### Socio-demographic predictors of negative health system consequences among moonlighters

Similar variations by province, sector, nursing category, and unit were found in the multiple regressions of health system consequences relevant to the moonlighting group only (Table 4).

**Table 4:** Multiple logistic regression of predictors of health system consequences

		Argued with doctors or other nurses	Conflicting schedules between primary and secondary jobs	Took vacation leave to do agency work or moonlighting	Took sick leave to do agency work or moonlighting	Stayed away without authority to do agency work or moonlighting	Treated patients differently in primary vs. secondary job	Any of these
								
Variable		OR (95% CI)	OR (95% CI)	OR (95% CI)	OR (95% CI)	OR (95% CI)	OR (95% CI)	OR (95% CI)
Province	Gauteng	–	–	–	–		–	–
	Eastern Cape	1.01	0.49	0.70	0.82		0.04**	0.79
		(0.58–1.75)	(0.23–1.05)	(0.32–1.55)	(0.31–2.17)		(0.00–0.36)	(0.45–1.40)
	Western Cape	0.45***	0.34***	0.46**	0.95		0.17*	0.45***
		(0.29–0.68)	(0.23–0.51)	(0.27–0.80)	(0.48–1.88)		(0.04–0.71)	(0.32–0.65)
	Free State	0.44***	1.01	1.12	0.24*		0.56	0.83
		(0.29–0.66)	(0.60–1.70)	(0.63–1.99)	(0.07–0.83)		(0.13–2.39)	(0.56–1.24)
Sex	Male		–	–				
	Female	0.82	0.73	0.68				
		(0.53–1.27)	(0.39–1.37)	(0.36–1.27)				
Age group	<25 years			–				–
	25–34 years			3.95				1.68
				(0.71–21.86)				(0.51–5.53)
	35–44 years			4.08				1.51
				(0.74–22.46)				(0.49–4.64)
	45–54 years			2.69				1.02
				(0.46–15.71)				(0.36–2.89)
	55+ years			2.18				0.99
				(0.36–13.29)				(0.28–3.48)
Sector	Public	–		–	–	–	–	–
	Private	1.16		1.09	0.44*	0.63	0.26	1.01
		(0.75–1.79)		(0.69–1.71)	(0.21–0.95)	(0.15–2.67)	(0.05–1.32)	(0.70–1.45)
	Agency	0.54*		0.20***	0.72	5.44*	0.44	0.49*
		(0.32–0.93)		(0.10–0.39)	(0.28–1.88)	(1.31–22.63)	(0.07–2.99)	(0.28–0.85)
Nursing category	Professional nurse	–		–	–			–
	Enrolled nurse	0.65		0.61*	1.66			0.69
		(0.38–1.13)		(0.40–0.94)	(0.75–3.68)			(0.47–1.01)
	Nursing assistant	0.86		0.28**	0.46			0.79
		(0.50–1.47)		(0.13–0.62)	(0.18–1.18)			(0.51–1.22)
Unit	General wards		–	–				–
	Maternity		0.64	0.94				0.70
			(0.23–1.75)	(0.41–2.15)				(0.42–1.19)
	ICU		1.08	2.76**				1.32
			(0.56–2.06)	(1.32–5.78)				(0.88–1.98)
	Theatre		1.47	1.52				1.69**
			(0.76–2.82)	(0.72–3.20)				(1.23–2.32)
	Other		0.59	1.75				1.15
			(0.30–1.16)	(0.88–3.51)				(0.81–1.62)
Years working at	Less than 1 year	–		–	–			–
primary job	1–4 years	1.67		0.88	0.72			1.16
		(0.87–3.21)		(0.48–1.59)	(0.25–2.11)			(0.71–1.88)
	5–9 years	1.40		1.04	0.44			1.38
		(0.92–2.11)		(0.49–2.24)	(0.10–1.86)			(0.88–2.17)
	10–14 years	1.75*		1.17	0.67			1.42
		(1.01–3.02)		(0.51–2.71)	(0.12–3.70)			(0.85–2.38)
	15–19 years	1.86*		1.03				1.41
		(1.07–3.24)		(0.45–2.34)				(0.78–2.56)
	20 or more years	1.35		1.05	0.11			1.23
		(0.79–2.29)		(0.56–1.97)	(0.01–1.06)			(0.75–2.03)
Constant		0.28***	0.19***	0.07**	0.08***	0.01***	0.05***	0.42
		(0.15–0.52)	(0.08–0.48)	(0.01–0.49)	(0.03–0.20)	(0.01–0.02)	(0.03–0.07)	(0.12–1.45)
Observations		1208	1299	1195	1135	1280	1280	1205
Model *p* value		<0.001	<0.001	<0.001		0.030	0.005	<0.001

Type 2 indicators in moonlighting nurses.

*** *p*<0.001

** *p*<0.01

* *p*<0.05.

In terms of geographical location and relative to Gauteng, those moonlighters from the Western Cape were less likely to report that they had conflicting schedules between primary and secondary jobs (OR: 0.34: 95% CI: 0.23–0.51); taken vacation leave to do agency work or moonlighting (OR: 0.46; 95% CI: 0.27–0.80); treated patients differently in the primary compared to the secondary job (OR: 0.17; 95% CI: 0.04–0.71) or argued with doctors or other nurses (OR: 0.45; 95% CI: 0.29–0.68). Participants from the Free State province were also less likely to report that they had taken sick leave to do agency work or moonlighting or that they argued with doctors or other nurses (Table 4).

Those working for a commercial nursing agency were more likely to report that they stayed away without authority to do agency work or moonlighting, with an odds ratio of 5.44 (95% CI: 1.31–22.63).

Relative to professional nurses, enrolled nurses, and nursing assistants were less likely to report that they had taken vacation leave to do agency work or moonlighting (Table 4).

## Discussion

In this study, 4 in 10 nurses reported moonlighting or working for a nursing agency in the year preceding the survey. South Africa's 5-year plan on human resources for health notes that ‘[i]t is common knowledge that public sector professionals “moonlight”, with or without permission, and that this reduces their productivity significantly and is a contributor to poor quality care’ (40, p. 51). Although this logic makes sense, our analysis did not find consistent statistically significant differences in self-reported health system incidents between moonlighting and non-moonlighting nurses. The bivariate analysis found that moonlighters were more likely to take sick leave when not sick (12.5% vs. 9.7%; *p*=0.011) and pay less attention to nursing work while on duty (13.2% vs. 10.3%, *p*=0.035). However, these differences did not remain statistically significant after adjusting for other socio-demographic variables in the multiple regression analysis, even though the odds ratios for these two variables were greater than 1 for moonlighters compared to non-moonlighters (Table 3).

Although the differences in these outcomes were not large enough to achieve statistical significance, it does not mean that the potential problems associated with the casualisation of the nurse workforce should be ignored in practice by hospital managers and health policy-makers. These reported health system incidents, which include taking sick leave when not sick and paying less attention to nursing work on duty, are serious. Cautionary evidence has been found in other studies. In the United Kingdom, for example, research has found that the use of temporary nursing staff (though moonlighting or agency nurses) contributes to the fatigue and burnout of permanent staff, who have to cover for or assist these temporary nurses; reduces the quality of patient care; and increases the risk of liability (27). Our study did not measure patient outcomes, but a US study also found that nursing homes that used a greater proportion of contract licensed staff were more likely to receive the worst quality deficiency ratings (29). Interestingly, the National Audit Office in the United Kingdom found that ward staff do not always report poor performance by temporary nursing staff but make sure that they do not return, hence the poor performance could be repeated elsewhere (27).

Although there is increasing policy attention to the performance of the health workforce (2, 40), the importance of dealing with fatigue among nurses appears to be a low policy priority. The finding that 51.5% of South African nurses reported that they felt too tired to work while on duty is alarming and has major implications for quality of patient care. Although not directly comparable because of different methodologies and tools used, the finding in our study is higher than those of a multicountry study where 38.1% of hospital nurses in China and 30.3% of nurses in Europe reported emotional exhaustion (8). There is well-documented evidence that nurse fatigue is a risk to patient safety and nurse well-being and contributes to negative patient outcomes and reduced job performance (8–10, 46–48). In recognition of this risk, the Registered Nurses Association of Ontario has published extensive guidelines on the prevention and mitigation of fatigue among nurses (48), and the American Institute of Medicine has highlighted the negative effects of fatigue on health care provider performance (49).

In the bivariate analysis, factors associated with nurses reporting feeling tired at work were geographical location (province), age group, marital status, public sector employment, category of nurse, and the number of years qualified as a nurse (Table 1). Interestingly, fewer moonlighters (50.5%) reported fatigue compared to non-moonlighters (52.2%). This unexpected result could be because hospital nursing managers tend to allocate fewer responsibilities to agency (moonlighting) nurses, preferring more complex nursing tasks (administration of intravenous medication) to be performed by permanent staff (50). In the multiple regression analysis, province, age younger than 35 years, public sector employment, and professional nursing category were predictors of feeling tired (Table 3). Study participants from the Eastern Cape Province and Gauteng were more likely to report feeling tired, compared to the other two provinces. Although a possible explanation for the higher rates in the Eastern Cape could be staff shortages in this more rural province with a high number of reported vacancies (40) and possibly larger workloads, more research is needed to determine the reasons for the observed provincial variation. Surprisingly, nurses older than 55 years were less likely to report feeling tired. The finding that public sector nurses were more likely to report feeling tired is not surprising in light of high patient numbers and workloads in the public sector, as the majority of South Africans are dependent on public hospitals for in-patient care (51). This is despite the fact that the same survey found higher moonlighting rates among private sector nurses, compared to those in the public sector (42). Professional nurses were also more likely to report feeling too tired to work while on duty. This could be because of their more advanced nursing skills and greater demand for their services in hospitals compared to other categories of nurses. Furthermore, professional nurses reported higher moonlighting or agency nursing rates, compared to other nursing categories (42).

The reported fatigue among nurses is exacerbated by other negative health system consequences found in this study. Among moonlighting nurses, 11.9% indicated that they had used their vacation leave to do agency work or moonlighting, contributing to fatigue. Nurses also reported unacceptably high rates of unauthorised absences leading to further understaffing, overwork, and health worker exhaustion. Of all nurses, 10.9% indicated that they had taken sick leave when not actually sick, and 5.6% had missed work without permission. These incidents were more common among moonlighting nurses who indicated that the unauthorised absences were sometimes used to do agency work or moonlighting.

A minority of nurses in the study (2.9%) reported a medico-legal incident, with category of nurse being the main predictor of reporting such an incident (Table 3). Nursing assistants were less likely to report a medico-legal incident (OR: 0.50; 95% CI: 0.26–0.93) relative to professional nurses (Table 3), again reflecting their much lower skills and type of tasks performed.

The study found that 33.7% of those who had done moonlighting or agency nursing were involved in any of the negative incidents (Table 2). There were provincial variations, which could be related to more effective management of moonlighting and agency nursing in the Western Cape and Free State. At the time of the study, Western Cape was one of two provinces that had a dedicated nursing director at the provincial level, who was tasked with the responsibility of standardisation of nursing policies, support, and monitoring of all health facilities.

There are a number of limitations of the study. As with all cross-sectional surveys, the temporal sequence between moonlighting or agency nursing and health system consequences could not be determined, leading to uncertainty as to whether these proxy indicators were causally related to moonlighting. Also, the consequences were self-reported, and we did not have objective measures of leave taken, absenteeism (staying away without authority), or medico-legal incidents. We also did not use a pretested instrument to measure fatigue. With self-reported data there is also always the possibility of social desirability bias resulting in lower disclosures of moonlighting or of negative health system consequences. The fact that the questionnaires were self-administered and anonymous provided greater privacy, which should have led to more accurate reporting of practices that are subject to social sanction. However, if moonlighting nurses were less likely to report health system consequences than non-moonlighters, it may also explain the lack of consistent statistical differences between the groups. Other limitations of the general survey are discussed in more detail in the previous article (42).

Notwithstanding these limitations, this study makes a number of important contributions. Our study is one of the first representative studies in South Africa and in Africa to examine the health system consequences of agency nursing and moonlighting – examples of casual or contingent work. The self-reported information on nurse fatigue in this large survey provides a basis for future comparisons of this aspect, which is a risk factor to patient safety and nurse well-being. The study also assisted in putting moonlighting and agency nursing on the health policy agenda in South Africa. However, further research is needed to assess the impact of moonlighting and agency nursing on more objective measures of nurse performance and ultimately on patient outcomes.

Our research has implications for health workforce policies and management and for quality of care. In South Africa, the Basic Conditions of Employment Act regulates the number of hours of employment in both the public and private health sectors (52), hence the legal framework is in place to prevent nurse fatigue. The Canadian guidelines propose the prevention and management of fatigue by allocating financial resources for infrastructure that enables health professionals to rest, recruitment and additional training facilities, appointment of additional staff, and education of all nurses about the causes and consequences of fatigue (48). South Africa's ‘Strategic Plan for Nursing Education, Training and Practice’ (41) contains a comprehensive set of recommendations that includes positive practice environments but there has been little, if any, implementation of these recommendations. Although the appointment of a Chief Nursing Officer at the beginning of 2014 is encouraging, a lot of effort is needed to overcome the implementation inertia characteristic of policy-making in South Africa (53).

In terms of moonlighting, the South African Public Service Act stipulates the conditions for additional, paid employment in the public sector (54). In theory, approval for moonlighting should only be granted if it does not impede the effective or efficient performance of the employee and, once approval is granted, implementation requires careful monitoring (40). The provincial health departments have recognised that the legal provisions are being ‘widely abused and should be much more closely managed’ (40, p. 59). Geographical location (province) explained some of the variation for the negative health system consequences, suggesting that there was better nursing management in some of the provinces. Mitigating the potential health system consequences of agency nursing and moonlighting requires decisive leadership and proactive management from the Chief Nursing Officer and hospital and nursing managers in both the public and private health sectors, rather than more legal provisions. At the same time, the national nursing association should spearhead a broader discussion on agency nursing and moonlighting and its implications for both patients and nurses. Best practice guidelines, drawing on the experience of other countries, should be developed for nurses and health facilities (33, 55).

Lastly, South Africa's emphasis on patient safety and quality of care (56) necessitates that agency nursing and moonlighting be addressed as part of the countrywide initiatives to create a quality revolution in health care.

## Conclusion

This study has investigated the negative health system consequences of agency nursing and moonlighting using a number of self-reported proxy indicators. Although we did not find consistent statistically significant differences between moonlighting and non-moonlighting nurses, the reported health system incidents are serious and further research is warranted. Although the process is complex, the casualisation of the nursing workforce cannot be viewed in isolation of South Africa's overall health system challenges, in particular its human resource challenges. In both the public and private health sectors, agency nurses are used to address nursing shortages (57). At the same time, casualisation with its concomitant shifting work patterns, an ageing nursing workforce, and a disjuncture between policies and implementation exacerbates nursing shortages. Although temporary nursing staff plays a role in dealing with actual and perceived nursing shortages, the potential negative consequences of agency nursing and moonlighting need to be counteracted through a combination of strong nursing leadership, effective management, and consultation with and buy-in from front-line nurses.
